# Phase II Clinical Trial of Pembrolizumab in Patients with Progressive Metastatic Pheochromocytomas and Paragangliomas

**DOI:** 10.3390/cancers12082307

**Published:** 2020-08-16

**Authors:** Camilo Jimenez, Vivek Subbiah, Bettzy Stephen, Junsheng Ma, Denai Milton, Mingxuan Xu, Abdualrazzak Zarifa, Fechukwu Omolara Akhmedzhanov, Apostolia Tsimberidou, Mouhammed Amir Habra, Jordi Rodon Anhert, Siqing Fu, Aung Naing

**Affiliations:** 1 Department of Endocrine Neoplasia and Hormonal Disorders, The University of Texas MD Anderson Cancer Center, Houston, TX 77030, USA; MAHabra@mdanderson.org; 2 Department of Investigational Cancer Therapeutics; The University of Texas MD Anderson Cancer Center, Houston, TX 77030, USA; vsubbiah@mdanderson.org (V.S.); BAStephen@mdanderson.org (B.S.); MXu2@mdanderson.org (M.X.); AZarifa@mdanderson.org (A.Z.); FOAkhmedzhanov@mdanderson.org (F.O.A.); atsimber@mdanderson.org (A.T.); jrodon@mdanderson.org (J.R.A.); siqingfu@mdanderson.org (S.F.); anaing@mdanderson.org (A.N.); 3 Department of Biostatistics; The University of Texas MD Anderson Cancer Center, Houston, TX 77030, USA; JMa4@mdanderson.org (J.M.); DRMilton@mdanderson.org (D.M.)

**Keywords:** PD-1 inhibition, pembrolizumab, metastatic pheochromocytoma, metastatic paraganglioma, pseudohypoxia

## Abstract

Metastatic pheochromocytomas and paragangliomas (MPPGs) are rare endocrine malignancies that are associated with high rates of morbidity and mortality because of their large tumor burden and location, progression, and release of catecholamines. Systemic therapies for MPPGs are limited. MPPGs are characterized by pseudohypoxia that may prevent immune system recognition. We conducted a phase II clinical trial of pembrolizumab in patients with progressive MPPGs. The primary endpoint was the non-progression rate at 27 weeks. The secondary endpoints included the objective response and clinical benefit rates, progression free and overall survival duration, and safety. We also determined whether PDL-1 expression and the presence of infiltrating mononuclear inflammatory cells in the primary tumor were associated with clinical response and hereditary background. Eleven patients were included in this trial, four (36%) with germline mutations and seven (64%) with hormonally active tumors. Four patients (40%, 95% confidence interval (CI) 12–74%) achieved the primary endpoint. The objective response rate was 9% (95% CI: 0–41%). The clinical benefit rate was 73% (95% CI: 39–94%). Four patients had grade 3 adverse events related to pembrolizumab. No patients experienced grade 4 or 5 adverse events or a catecholamine crisis. Progression free survival time was 5.7 months (95% CI: 4.37—not reached). The median survival duration was 19 months (95% CI: 9.9—not reached). PDL-1 expression and the presence of infiltrating mononuclear inflammatory cells in the primary tumor did not seem to be associated with disease response. Single-agent pembrolizumab has modest treatment efficacy in patients with progressive MPPGs. Positive responses seemed to be independent of patients’ hereditary backgrounds, tumor hormonal status, and the presence of infiltrating mononuclear inflammatory cells or PDL-1 expression in the primary tumor.

## 1. Introduction

Pheochromocytomas and paragangliomas are rare neuroendocrine tumors. Pheochromocytomas originate in the adrenal medulla, and paragangliomas originate in the parasympathetic and sympathetic autonomic nervous system ganglia. Pheochromocytomas and sympathetic paragangliomas frequently release excessive amounts of catecholamines, predisposing patients to cardiovascular and gastrointestinal disease [1,2]. Most patients with pheochromocytomas and paragangliomas present with localized tumors that are curable with surgery. However, 15–20% of these tumors are associated with metastases [3]. Unfortunately, there are no histological, molecular, genetic, or biochemical markers that can be used to predict the malignant potential of the primary tumor. Therefore, the definition of malignancy relies on the presence of metastases [4].

Metastases are mainly found in the lymph nodes, skeleton, lungs, and liver, and most patients with malignancy present with stage IV disease that compromises their survival [5,6]. Only 30–60% of patients with distant metastases live 5 years after their initial diagnosis [6,7]. The high morbidity and mortality rates of patients with metastatic pheochromocytomas and paragangliomas (MPPGs) are related to the tumor burden and location, the speed of disease progression, and catecholamine-mediated endocrine complications [8]. Survival curves demonstrate that MPPGs exhibit heterogeneous behavior [6]. Some tumors are aggressive, while others exhibit minimal or no progression over time. Patients with indolent MPPGs may have normal life spans and not require systemic therapy [9]; however, systemic therapies for patients with progressive MPPGs are limited.

High-specific activity iodine-131 meta-iodine benzyl guanidine (HSA-I-131-MIBG) recently became the first medication to be approved by the United States Food and Drug Administration for the treatment of MIBG-avid MPPGs [10]. Approval was granted on the basis of the impressive clinical benefit rate (CBR), as demonstrated by a pivotal phase II clinical trial. Although the medication was not associated with tumor elimination, patients achieved partial responses (PRs) and prolonged disease stabilization. Currently, HSA-I-131-MIBG is the only systemic therapy that has been approved for patients with MPPGs. However, it is not suitable for patients with MPPGs that do not express the cell membrane norepinephrine transporter [11,12]. Systemic chemotherapy (mainly a combination of cyclophosphamide, dacarbazine, and vincristine) has been used to treat MPPGs since 1985. Chemotherapy is associated with tumor shrinkage, symptom improvement, and possibly longer survival duration [13]. However, only 37% of patients experience a response to chemotherapy and it results in overwhelming toxicity [14]. Ongoing clinical trials are evaluating tyrosine kinase inhibitors (e.g., cabozantinib) and peptide receptor radionuclide therapy (e.g., 177-Lutetium-dotatate). Preliminary results have shown that antiangiogenic therapies may lead to tumor size reduction, disease stabilization, and symptom improvement in a substantial number of patients; however, most MPPGs acquire resistance over time [15]. Initial trials with 177-Lutetium-dotatate showed limited responses; however, these trials only included a small number of patients [16,17]; recent observations suggest that some patients may indeed benefit from this medication [18,19]. Therefore, other therapies must be investigated.

Approximately 30–50% of patients with MPPGs carry germline mutations of the succinate dehydrogenase subunit B (*SDHB*) gene [20]. These mutations predispose patients to tumor pseudohypoxia, abnormal tumor cell replication, and tumor necrosis and angiogenesis and may prevent immune system recognition [21]. Of interest, a substantial number of apparently sporadic MPPGs have a similar molecular profile as that of *SDHB* (+) tumors [22]. Deregulation of cellular energetics is a universal hallmark of cancer that interferes with T cell effector function, causes immune suppression and tolerance, impairs tumor infiltration by T cells, and induces resistance to cytotoxic T cells [23]. T cells express the programmed cell death-1 (PD-1) immune checkpoint receptor; this receptor binds the programmed cell death ligands 1 and 2 (PDL-1; PDL-2) that are expressed by the tumor microenvironment, preventing the immune surveillance of tumor cells. A recent seminal study found that a substantial number of MPPGs express the programmed cell death ligands, shaping the tumor’s immune-tolerogenic micro-environment [24]. Pembrolizumab is a humanized IgG4κ monoclonal antibody; it targets the PD1/PDL-1 pathway that is often hijacked by cancer cells to escape immune surveillance. Pembrolizumab is specifically an immune check-point inhibitor of PD1 and has been approved for the treatment of several malignancies.

We previously reported the results of an interim analysis of a phase II study of pembrolizumab in patients with rare tumors [25]. Anti-tumor activity was observed in patients with adrenal cortical carcinoma, squamous cell carcinoma, cancer of unknown origin, and MPPGs. We now report the final results of the phase II study in patients with MPPGs. The primary endpoint was the non-progression rate at 27 weeks. The secondary endpoints included the objective response and clinical benefit rates, overall survival duration, and safety. We also determined whether PDL-1 expression and the presence of infiltrating mononuclear inflammatory cells in the primary tumor were associated with clinical response and hereditary background.

## 2. Patients and Methods

### 2.1. Patients

All patients were recruited from 15 August 2016, through 1 March 2020 from The University of Texas MD Anderson Cancer Center (Houston, TX, USA). Participants were at least 18 years of age and had a histopathological diagnosis of non-resectable MPPG and radiographic confirmation of metastases with 6 months of disease progression prior to enrollment. Progression was determined by radiographic studies using the Response Evaluation Criteria in Solid Tumors (RECIST) version 1.1. Patients were included if they had an Eastern Cooperative Oncology Group (ECOG) performance status <2 and a life expectancy >6 months, as determined by the principal investigator. All patients had adequate organ function and measurable disease. The clinical trial allowed the participation of therapy-naïve patients as well as those who had been previously treated with chemotherapy, radiopharmaceuticals, or tyrosine kinase inhibitors. Patients who had been previously treated with anti-PD1, anti-PD-L1, or anti-PD-L2 medications were excluded from this trial.

### 2.2. Study Design

This was an open-label, investigator-initiated phase II trial of pembrolizumab in patients with progressive MPPGs. Although the expression of PDL-1 in patients with non-small cell lung cancer is typically associated with a positive immunological response [26], this association is not clear in patients with other cancers [27]. In this phase II study, we evaluated the possible association between MPPG PDL-1 expression and therapeutic response. Subsequently, patients with MPPGs were enrolled regardless of tumor PD-L1 expression.

The primary objective was to identify early indications of efficacy by evaluating the non-progression rate at 27 weeks (nine cycles). The non-progression rate was defined as the number of patients who were alive and progression free at 27 weeks, as determined by immune-related RECIST (irRECIST). The secondary objectives included the (1) objective response rate (ORR), defined as the percentage of patients with a complete response (CR) and PR, as per irRECIST; (2) CBR, defined as the percentage of patients with CR, PR, and stable disease (SD) ≥4 months, as per irRECIST; (3) safety and tolerability of pembrolizumab; and (4) progression free and overall survival rates. We also determined whether PDL-1 expression and the presence of infiltrating mononuclear inflammatory cells in the primary tumor were associated with clinical response and hereditary background.

The protocol was approved by MD Anderson Institutional Review Board (IRB Registration Number: IRB 1 IRB00000121, approval date 15 June 2016) and the United States Food and Drug Administration. The study was conducted in accordance with the Declaration of Helsinki and the International Conference on Harmonization Good Clinical Practice guidelines. The trial was registered on ClinicalTrials.Gov (https://clinicaltrials.gov, ClinicalTrials.gov Identifier: NCT02721732). All patients signed informed consent before enrollment.

### 2.3. Intervention

Pembrolizumab was administered intravenously at a dose of 200 mg every 3 weeks. Treatment continued until the patient exhibited radiographic or clinical disease progression or unacceptable adverse events, withdrew from the trial, was non-compliant with trial requirements, or completed 24 months of treatment with pembrolizumab. Patients with a CR could discontinue pembrolizumab after undergoing at least 27 weeks of treatment.

### 2.4. Procedures

Patients underwent radiographic studies, including computed tomography scanning or magnetic resonance imaging, at baseline and every 9 weeks for the first 27 weeks (6 months) to assess response to treatment, according to irRECIST. For patients who experienced a CR, PR, or SD for longer than 27 weeks, computed tomography or magnetic resonance imaging was performed every 12 weeks thereafter. If initial radiographic studies revealed progressive disease (PD), imaging was repeated 4 weeks later to confirm progression. If repeated imaging showed a reduction in tumor size, treatment with pembrolizumab was continued for presumed pseudo-progression. If repeated imaging showed PD, treatment with pembrolizumab was discontinued. Investigators considered all target lesions and non-target lesions to determine whether the tumor had increased or decreased in size. For patients with predominant bone metastases, an FDG-PET computed tomography scan was also obtained. Response to treatment was evaluated by RECIST 1.1 to identify atypical responses.

Adverse events were evaluated as per the National Cancer Institute Common Terminology Criteria for Adverse Events version 4.03. Patients with hormonally active MPPGs were followed up using measurements of plasma metanephrines at the time of radiographic assessment. Blood pressure and pulse were documented at every visit.

For each patient, tumor specimens from archival tissue samples or newly obtained biopsy specimens (if archival tissue was not available) were evaluated by immunohistochemical analysis to determine the expression of PDL-1 in tumor cells and tumor-infiltrating mononuclear inflammatory cells by Qualtek (QualTek Molecular Laboratories, Goleta, CA, USA). We used the Merck 22C3 antibody (Merck & Co., Inc., Kenilworth, NJ, USA) for PDL-1 staining. The expression of PDL-1 was assessed and scored by a certified pathologist. An H-score of 0–300 was assigned to tumor samples. Using a recursive partitioning analysis, we identified a score of 42.5 as the optimal cut-off point for PDL-1 H-score [25]. To evaluate the presence of tumor-infiltrating lymphocytes (TILs), we performed a morphologic assessment of hematoxylin-eosin sections within the tumor nests. The expression of TILs was graded on the basis of their density from 0 to 3: A score of 0 for absence of TILs, a score of 1 for a few TILs, and a score of ≥2 for a high density of TILs.

### 2.5. Statistical Analysis

The trial used Simon’s optimal two-stage design with 12 patients planned in the first stage and 13 in the second stage. At least three of the first 12 treated patients had to be alive and progression free at 27 weeks before other patients could be enrolled. The null success rate was set at 20% and the alternative rate at 40%. This method has a 10% type I error rate, 82% power, and 56% probability of stopping after the first stage if the progression-free survival rate at 27 weeks is 20%.

Patients’ demographic characteristics were summarized using descriptive statistics of frequencies and percentages. The 27-week progression-free rate was estimated, along with the 95% confidence interval. Progression free survival time was computed from the first date of treatment to the date of irRECIST progression or death as a 1st event, with censoring at last clinical evaluation if no progression free survival event was observed. Overall survival time was computed from the first date of treatment to death date, where patients who were alive at last follow up date were administratively censored. Unadjusted survival distributions were estimated by the Kaplan-Meier method. ORR and CBR were reported with 95% confidence intervals. A waterfall plot was used to illustrate the maximum percentage change in tumor measurement per irRECIST compared with the baseline. The duration of response was defined as the period of time between the date of the first response and the date of disease progression. A swimmer plot was used to illustrate time on treatment and duration of response. All patients who had at least one adequate on-study radiographic assessment were included in the final tumor outcome analysis. All patients who had received at least one dose of pembrolizumab were included in the final toxicity analysis.

Treatment-related adverse events were summarized as the number and percentage of patients with adverse events that were described as possibly, probably, or definitely related to treatment. In the event that a patient experienced repeated episodes of the same adverse event, the event with the highest severity or strongest causal relationship to pembrolizumab were used for tabulations. Patients who discontinued participation in the study before 27 weeks for reasons other than PD or death were considered to be not evaluable for the primary endpoint. All statistical analyses were performed using R software version 3.5.1 (R Foundation for Statistical Computing, Vienna, Austria).

## 3. Results

### 3.1. Patient Characteristics

Eleven patients with progressive MPPGs were enrolled in our study; their demographic and clinical characteristics are shown in Table 1. The patients’ median age at the time of enrollment was 53.6 years (range = 22.7–74.4 years). Seven patients were male (64%). The median size of the primary tumor was 7 cm (range = 2.8–11.3 cm). Most primary tumors were located in the abdominal cavity. Seven patients (64%) had tumors that secreted excessive amounts of norepinephrine. Seven patients (70%) had tumors that expressed the norepinephrine transporter (MIBG+). Four patients (36%) had a germline mutation; two had paraganglioma syndrome type 4 (*SDHB*), one had a paraganglioma syndrome type 1 (*SDHD*), and one had Lynch syndrome (*PMS2*). Three patients were naïve to systemic therapy; all other patients were treated with at least one type of systemic therapy prior to participating in the clinical trial. All patients had Eastern Cooperative Oncology Group status <2.

### 3.2. Efficacy

**Figure 1:**
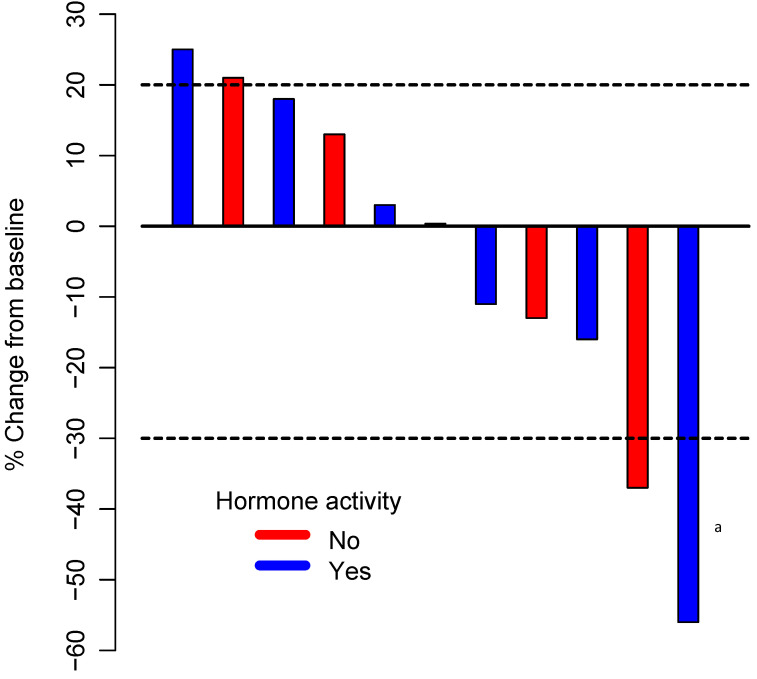
Waterfall plot illustrating the best response to pembrolizumab therapy in 11 patients. The area below the lower dotted line represents a partial response (30% decrease in the sum of diameters of target lesions compared with the baseline); the area between the two dotted lines represents stable disease; and the area above the upper dotted line represents progressive disease (20% increase in the sum of the diameters of target lesions compared with the smallest sum during the study), based on immune-related Response Evaluation Criteria in Solid Tumors. ^a^ Patient with progressive disease (unconfirmed partial response).

All patients who were enrolled in this trial received at least one dose of pembrolizumab. One patient experienced toxicity before week 27 and was therefore excluded from the primary outcome analysis (but included in all other analyses). Among the remaining 10 patients, four were alive, with no evidence of progression, at 27 weeks. The estimated 27-week progression-free rate was 40% (95% CI: 12–74%). The distribution of the best response by irRECIST was one irPR (9%); seven irSD (64%), including three patients with some degree of regression; and three irPD (27%). The ORR was 9% (95% CI: 0–41%), and the CBR was 72% (95% CI: 39–94%). Figure 1 illustrates the tumor percentage change from the baseline for the best response for all participants in the study.

One patient with a progressive sympathetic paraganglioma that was metastatic to the skeleton, lymph nodes, liver, and lungs and associated with excessive release of noradrenaline had been previously treated with cabozantinib, resulting in a PR. Eight months later the patient experienced disease progression and discontinued treatment. At the time of progression, the patient was found to have grade 3 hand and foot syndrome, complicated by an infection with *Pseudomona aeruginosa*. The patient received antibiotics for 6 weeks. In the interim, the tumor rapidly progressed. On physical examination, the patient had developed visible, firm, fixed left supraclavicular and axillary metastatic lymphadenopathy conglomerates; radiographic studies revealed new and progressive metastatic lesions in the liver, lungs, and skeleton. The patient’s symptoms of catecholamine excess were exacerbated, necessitating the addition of three antihypertensives. Four days after starting pembrolizumab, the patient became orthostatic, and the antihypertensives were discontinued. The cervical and axillary lymphadenopathy conglomerates were no longer palpable. Radiographic studies revealed the disappearance of some lesions in the lungs, liver, and lymph nodes and a substantial reduction in target lesion size (56% decrease from baseline by irPR). The symptoms of catecholamine excess subsided, and the plasma normetanephrine level rose from 54 to 236 nmol/L (normal <0.90 nmol/L). We believe that the pronounced elevation of plasma normetanephrine concentrations was caused by the rapid tumor destruction induced by pembrolizumab. However, we cannot explain why the symptoms of catecholamine excess subsided almost immediately despite these high concentrations. The patient developed grade 3 liver toxicity. Pembrolizumab was discontinued. Liver function normalized 6 weeks later; at the time of reassessment, the patient had a new liver lesion despite a substantial reduction in other lesions; as per trial design, the patient discontinued participation because of PD. The patient had a non-confirmed PR.

### 3.3. Duration of Response and Overall Survival

**Figure 2:**
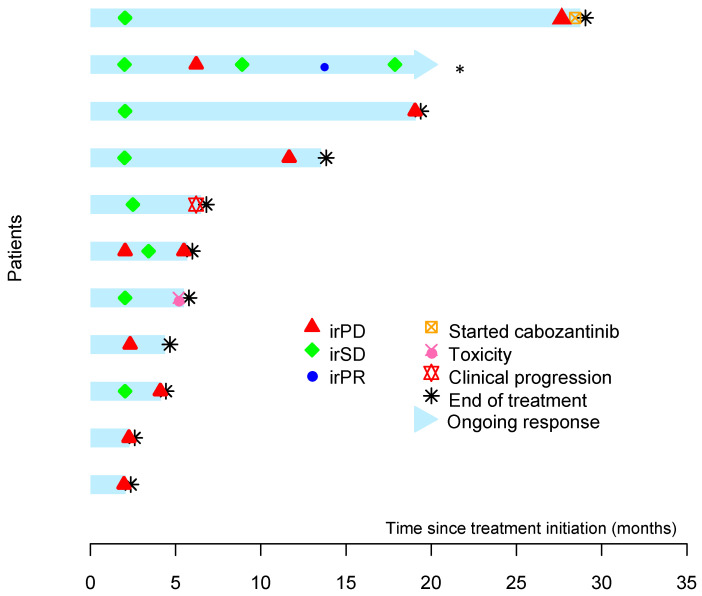
Swimmer plot of time to and duration of response in all patients treated with pembrolizumab. By the time of data cut-off, the second patient from the top was still experiencing a response *. The first patient from the top had stable disease for 24 months.

The median follow-up time was 17.9 months (IQR: 9.3–20.2 months). One patient discontinued treatment early because of toxicity. Seven patients exhibited radiographic evidence of disease progression, and one patient had clinical progression. One patient with a metastatic head and neck paraganglioma that was associated with a germline *SDHD* mutation experienced SD for 24 months; during the last month of therapy with pembrolizumab, the patient developed a pathologic fracture of the spine that was related to tumor progression. Pembrolizumab was discontinued. The patient started cabozantinib and is responding to this drug. The patient with the confirmed PR is still on active treatment with pembrolizumab. Figure 2 illustrates the time to and duration of response for all patients.

**Figure 3:**
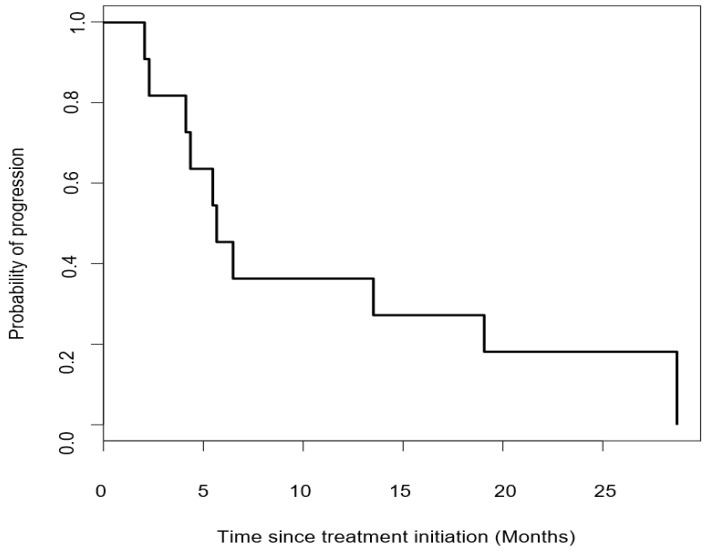
Kaplan-Meier curve of progression free survival in patients treated with pembrolizumab. The median progression free survival time was 5.7 months (95% CI: 4.37—not reached).

Six patients died because of disease progression. The median progression free survival time was 5.7 months (95% CI = 4.37—not reached) (Figure 3).

**Figure 4:**
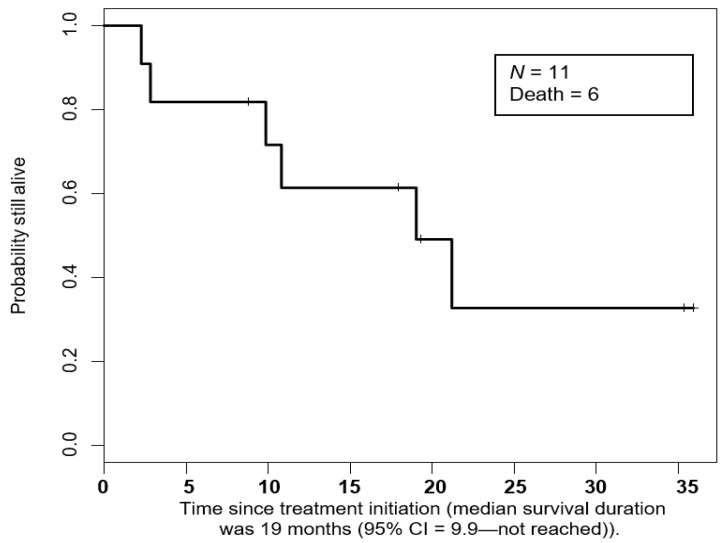
Kaplan-Meier curve of overall survival duration in patients treated with pembrolizumab.

The median survival duration was 19 months (95% CI = 9.9—not reached) (Figure 4).

### 3.4. Safety

Treatment-related adverse events of any grade occurred in all patients (total = 26 events) (Table 2). Elevation of liver enzymes and constitutional symptoms such as fatigue, lack of appetite, and dysgeusia were the most common related side effects. Most events were mild (grade 1) to moderate (grade 2). Grade 3 effects included asymptomatic elevation of the liver enzymes in one patient and anemia in two patients. One patient experienced an immune-related right eye III cranial nerve palsy (myasthenia gravis like); this patient discontinued participation in the study. In total, six adverse events were immune related (23%). There were no grade 4 or 5 related adverse events. Patients did not experience grade 3 or higher hypertension; no patients had a catecholamine crisis.

### 3.5. PDL-1 Membrane Expression and TILs

Three patients had tumors that expressed PDL-1. The patient with the highest PDL-1 H-score (75) exhibited SD at 27 weeks. The patient experienced PD after 58 weeks of treatment with pembrolizumab. The second patient had a PDL-1 H-score of 73 and low TILs (score = 1). The patient could not continue on the trial beyond 24 weeks because of grade 3 immune-related toxicity (III cranial nerve palsy). The third patient had a PDL-1 H-score of 35 and did not meet the primary endpoint. The patient with a confirmed PR had no PDL-1 expression in baseline tumor samples; however, the primary tumor had high TILs (score = 3). Overall, there was no clear association between PDL-1 expression and TILs in the primary tumor and clinical response (Table 3).

## 4. Discussion

In this study, we report the final results of a phase II study of pembrolizumab in patients with MPPGs; to our knowledge, this was the first clinical trial of immunotherapy in this disease. Our results include the results of long-term follow-up evaluations, the final data on pembrolizumab efficacy, and the correlations between specific and inherent aspects of MPPGs that have not been reported previously. Four patients (40%) who were treated with pembrolizumab showed no evidence of progression 27 weeks after the treatment had started. Three of these patients had SD, and one had a confirmed PR. No patients experienced a CR. Overall, pembrolizumab was well tolerated. The most common side effect was mild fatigue, and no patients developed a catecholamine crisis during or immediately after the infusion of the drug. The results of this trial indicate that pembrolizumab has modest anti-neoplastic activity against MPPGs, with an acceptable safety profile.

In regards to the primary endpoint, the longest periods of stability were noted in two patients with non-hormonally active tumors. One patient had a head and neck paraganglioma that was metastatic to the liver, lungs, breast, lymph nodes, and skeleton in the context of paraganglioma syndrome type 1; the patient experienced no clinical or radiographic disease progression for 24 months and had a non-significant reduction in tumor size, as per irRECIST 1.1 (−11%). The second patient had an apparently sporadic tumor that was metastatic to the liver and lungs; this patient achieved a confirmed PR and continued to experience a response after 20 months of therapy. Of interest, these two patients had rapid PD before participating in this trial and had been previously treated with other systemic therapies. Therefore, their results are reassuring.

The two other patients who experienced the primary endpoint had tumors that caused hypertension as a result of the excessive release of noradrenaline. One of these patients experienced some tumor regression (–13%) and continued on stable antihypertensive therapy until disease progression. The other patient did not experience tumor size reduction, required higher doses of antihypertensives, and exhibited intensification of catecholamine excess symptoms over time with deterioration of quality of life; this patient discontinued participation because of clinical disease progression, despite radiographic evidence of tumor stability. In regards to the secondary endpoints, although the ORR was less than 10%, the CBR was 72%. Several of the patients with SD experienced non-significant tumor enlargement, as per RECIST 1.1 (<20%). Whether pembrolizumab slowed down the proliferative rate of some of these tumors or whether the unpredictable nature of this neuroendocrine disease was associated with slow tumor growth during the trial is still to be determined. To date, very few clinical trials in MPPGs have been published [10,28,29,30]. Of these, none have explored or consistently identified a lack of disease progression at 27 weeks as an important cut point. As a result, the non-progression rate in our trial should be interpreted with caution. 

Two patients experienced a substantial reduction in tumor size (>30%). As discussed above, one patient had a confirmed PR and continues to experience a response to therapy. The second patient carried an *SDHB* germline mutation and was treated with antibiotics for severe hand and foot syndrome that was complicated by an infection with *Pseudomona aeruginosa*. This patient had been previously treated with cabozantinib, a potent antiangiogenic tyrosine kinase inhibitor. With the first dose of pembrolizumab, the patient experienced rapid shrinkage or disappearance of several metastatic lesions, normalization of blood pressure, and remission of symptoms of catecholamine excess. However, the patient experienced severe liver toxicity, with a long recovery. Subsequent radiographic studies revealed a small new lesion in the liver; because of this, the patient discontinued participation in the study, even though other metastases had decreased in size or disappeared. This patient did not meet the primary study endpoint, and the response was categorized as PD. Clinical trials of pembrolizumab in patients with other malignancies have shown rapid tumor responses [31]. In our patient, prior exposure to cabozantinib could have led to tumor vascular normalization and enhanced immune system recognition [15,32]; however, other patients in this trial had also been previously treated with cabozantinib and did not experience dramatic responses to pembrolizumab. It is possible that the introduction of bacterial non-self antigens that mimic human self antigens, expanding T cells with high-affinity receptors, increases the efficiency of the immune system against MPPGs during exposure to pembrolizumab, which prevents negative immune feedback loops [33,34]. Novel clinical trials in different malignancies are exploring this hypothesis. Regulatory agencies are currently evaluating a clinical trial that would test the combination of the PDL-1 inhibitor nivolumab and microbially derived peptides that mimic human cell antigens in MPPGs.

MPPGs are associated with the highest rate of single germline mutations of any oncological disease; however, they have some of the lowest rates of somatic mutations among malignancies [35]. Thus, they are expected to be associated with low immunogenic antigen density and no or minimal inflammation or infiltration of T cells (cold tumors) [36]. The results of our trial indicate that indeed, some primary tumors exhibit no or minimal TILs; however, 44% of the primary tumors had high TIL density. This contrasts with the results of published studies of pheochromocytomas and paragangliomas. Previous analyses may have been performed in non-metastatic tumors; nevertheless, the presence of TILs does not seem to be predictive of the primary tumor’s metastatic potential or response to pembrolizumab. Similarly, PDL-1 expression in the primary tumor samples did not seem to be correlated with clinical response. The H-score of the tumor samples for PDL-1 expression was generally low (the highest was only 75); this could explain why the response to pembrolizumab was modest. However, the best responses were noted in patients with primary tumors that did not express PDL-1. Perhaps, metastatic lesions had high PDL-1 expression. Unfortunately, we were not able to assess the H-score for PDL-1 in those lesions.

Of interest, one patient in our study had Lynch syndrome. It is difficult to determine whether an association exists between a *PMS2* germline mutation and the development of MPPG. To the best of our knowledge, no prior cases of Lynch syndrome and MPPGs have been described. Patients with Lynch syndrome-related tumors seem to exhibit high response rates to immunotherapy [37,38]. However, the patient in our study discontinued participation because of a myasthenia gravis-like ocular toxicity and could not be evaluated for the primary endpoint.

The safety profile of pembrolizumab in patients with MPPGs is concordant with that reported in clinical trials of other malignancies [39]. Of interest, no side effects related to hormone release were noted in our trial. Nevertheless, all patients with hormonally active MPPGs should be treated with alpha-blockers, beta-blockers, or other antihypertensives in preparation for systemic therapy [28].

Our study has limitations. First, the patient sample was small. In addition, this was a single institution study of a rare tumor. In the United States, only 100–200 new MPPGs are diagnosed every year; as discussed earlier, many of these tumors are indolent and do not require systemic therapy. In addition, the recent FDA approval of HSA-I-131-MIBG led to this drug becoming the standard of care for MIBG-avid MPPGs. As a result, it was difficult to recruit patients for this trial. Second, the samples for analysis were mainly obtained from primary tumors; this may have influenced the expression of immune biomarkers. Although biopsies of metastases were encouraged by the protocol, concerns about a potential catecholamine crisis prevented us from performing additional studies. Third, it was not possible to determine metanephrine’s biochemical marker correlations as only a small number of samples were available for analysis. Fourth, it was not possible to perform a correlative statistical analysis because of the very small sample size. Nevertheless, the strength of our study is that it shows that pembrolizumab can be effective in some patients with MPPGs who have failed to experience a response to other therapies. However, we cannot recommend pembrolizumab alone as a first-line therapy for MPPGs.

## 5. Conclusions

Immunotherapy with the single agent pembrolizumab had modest efficacy in patients with MPPGs, regardless of their hormonal, hereditary, or PDL-1 status. Treatment was well tolerated, with a low rate of serious adverse events. Further investigation is needed into other factors that may contribute to the success or failure of immunotherapy, such as exposure to non-self antigens or combinations with other systemic therapies.

## Figures and Tables

**Table 1:** Patient characteristics.

Characteristic	*n* (%)
Sex	
Female	4 (36)
Male	7 (64)
Germline mutation	
No	7 (64)
*SDHB*	2 (18)
*SDHD*	1 (9)
*PMS2*	1 (9)
Primary tumor location	
Adrenal (pheochromocytoma)	4 (36)
Sympathetic abdominal paraganglia	6 (55)
Head and neck (parasympathetic)	1 (9)
Hormonal activity	
Yes	7 (64)
No	4 (36)
MIBG uptake	
Yes	7 (64)
No	3 (27)
Unknown	1 (9)
Prior systemic therapy	
Naïve	3 (28)
High-specific activity MIBG	1 (9)
Cabozantinib	1 (9)
CVD	1 (9)
CVD, cabozantinib, lenvatinib	1 (9)
Surgery, cabozantinib	2 (18)
Surgery, CVD	1 (9)
Surgery, CVD, cabozantinib	1 (9)
Eastern Cooperative Oncology Group	
0	4 (36)
1	7 (64)

*SDHB,* paraganglioma syndrome type 4; *SDHD,* paraganglioma syndrome type 1; *PMS2,* Lynch syndrome; MIBG, meta-iodine benzyl guanidine; CVD, cyclophosphamide, vincristine, dacarbazine.

**Table 2:** Treatment-related adverse events.

Adverse Event	Overall, *n* (%)	Grade 1, *n* (%)	Grade 2, *n* (%)	Grade 3, *n* (%)
Total	26	18	4	4
Laboratory abnormalities				
Increased ALT/AST	4 (16)	3 (17)	0 (0)	1 (25)
Increased alkaline phosphatase	3 (12)	3 (17)	0 (0)	0 (0)
Increased bilirubin	1 (4)	0	1 (25)	0
Increased creatinine	1 (4)	0	1 (25)	0
Anemia	5 (19)	2 (11)	1 (25)	2 (50)
General disorders				
Anorexia	1 (4)	1 (6)	0	0
Dysgeusia	1 (4)	0	1 (25)	0
Fatigue	3 (12)	3 (17)	0	0
Genital edema	1 (4)	1 (6)	0	0
Limb edema	2 (8)	2 (11)	0	0
Hyperthyroidism	1 (4)	1 (6)	0	0
Neurological disorders				
Peripheral sensory neuropathy	1 (4)	1 (6)	0	0
III cranial nerve paralysis	1 (4)	0	0	1 (25)
Rash macula-papular	1 (4)	1 (6)	0	0

ALT/AST, alanine aminotransferase/aspartate aminotransferase.

**Table 3:** Correlations between PDL-1 H-score, TILs, radiographic response, and survivorship in MPPGs.

Patient	NPR at 27 Weeks (Met or No Met)	PDL-1 H-Score	TILs	Best IrRECIST Response ^a^	Alive at Last Follow-Up
1	Met	75	1	SD (−13%)	Yes
2	Met	N/A	N/A	SD (−11%)	Yes
3	no Met	0	2	-	No
4	no Met	0	2	-	No
5	Met	0	0	SD ^b^	No
6	no Met	0	0	- ^c^	No
7	no Met	35	1	-	Yes
8	no Met	N/A	N/A	-	No
9	Met	0	3	PR (−37%)	Yes
10	no Met	0	3	-	No
11	Not evaluable	73	1	-	Yes

PDL-1 H, programmed cell death ligand-1; TIL, tumor-infiltrating lymphocyte; MPPG, metastatic pheochromocytomas and paraganglioma; NPR, non-progression rate; Met:, patients achieve the primary endpoint; No met, patients did not achieve the primary endpoint; IrRECIST, immune-related Response Evaluation Criteria in Solid Tumors; SD, stable disease; N/A, not available; PR, partial response. ^a^ in patients who met the primary endpoint, ^b^ clinical progression, ^c^ unconfirmed PR (–56%).
